# Chemotherapy-Induced Regression of an Adrenocorticotropin-Secreting Pituitary Carcinoma Accompanied by Secondary Adrenal Insufficiency

**DOI:** 10.1155/2013/675298

**Published:** 2013-12-22

**Authors:** Robert Frank Cornell, Daniel F. Kelly, Gal Bordo, Ty B. Carroll, Huy T. Duong, Julie Kim, Yuki Takasumi, James P. Thomas, Yee Lan Wong, James W. Findling

**Affiliations:** ^1^Division of Hematology and Oncology, Department of Medicine, Vanderbilt University Medical Center, Nashville, TN 37232, USA; ^2^Brain Tumor Center & Pituitary Disorders Program, John Wayne Cancer Institute at Saint John's Health Center, Santa Monica, CA 90404, USA; ^3^Endocrinology Center and Clinics, Department of Medicine, The Medical College of Wisconsin, Milwaukee, WI 53226, USA; ^4^Division of Hematology and Oncology, Department of Medicine, The Medical College of Wisconsin, Milwaukee, WI 53226, USA

## Abstract

*Purpose*. Adrenocorticotropin- (ACTH-) secreting pituitary carcinomas are rare and require multimodality treatment. The aim of this study was to report the response to various therapies and discuss the potential development of secondary adrenal insufficiency with cytotoxic chemotherapy. *Methods*. This report describes a man with a large silent corticotroph adenoma progressing to endogenous hypercortisolism and metastatic ACTH-secreting pituitary carcinoma over a period of 14 years. *Results*. Seven years after initial presentation, progressive tumor enlargement associated with the development of hypercortisolism mandated multiple pituitary tumor debulking procedures and radiotherapy. Testing of the Ki-67 proliferation index was markedly high and he developed a hepatic metastasis. Combination therapy with cisplatin and etoposide resulted in a substantial reduction in tumor size, near-complete regression of his liver metastasis, and dramatic decrease in ACTH secretion. This unexpectedly resulted in symptomatic secondary adrenal insufficiency. *Conclusions*. This is the first reported case of secondary adrenal insufficiency after use of cytotoxic chemotherapy for metastatic ACTH-secreting pituitary carcinoma. High proliferative indices may be predictive of dramatic responses to chemotherapy. Given the potential for such responses, the development of secondary adrenal insufficiency may occur and patients should be monitored accordingly.

## 1. Introduction

Pituitary carcinomas are rare, constituting less than 1% of patients with pituitary tumors [1]. Aggressive pituitary tumors are characterized by invasion of the parasellar region including the cavernous sinus, bone, and subarachnoid space of the suprasellar region. The diagnosis of pituitary carcinoma usually requires evidence of either intracranial or extracranial metastases [2]. Adrenocorticotropin- (ACTH-) secreting pituitary tumors are the most common secretory subtype which undergo malignant transformation [3, 4].

The majority of ACTH-secreting pituitary carcinomas present with clinical and biochemical features of Cushing's syndrome and can occasionally manifest after bilateral adrenalectomy for Cushing's disease (Nelson's syndrome) [4]. However, some corticotroph carcinomas develop from “silent” corticotroph adenomas which secrete precursors of ACTH from the prohormone proopiomelanocortin (POMC) detected in ACTH immunoassays. Some of these “silent” corticotroph tumors may undergo malignant transformation to elaborate biologically active ACTH and endogenous hypercortisolism may ensue [3].

Pituitary carcinomas are notoriously difficult to treat. Surgical debulking of the tumor is considered primary therapy and may help alleviate symptoms associated with perisellar invasion and attenuate the hormone hypersecretory state. Radiotherapy (conventional, proton beam, and gamma knife) is another therapeutic option. Although anecdotal reports have suggested a delay in tumor progression with radiotherapy, the rarity of pituitary carcinoma and its variable natural history make it difficult to assess the degree of benefit. Many single agent and combination chemotherapy regimens have been utilized with limited responses. The most commonly reported cytotoxic drugs used in pituitary carcinomas have been lomustine (CCNU) and 5-fluorouracil (5FU). More recently, temozolomide (TMZ), an oral alkylating agent approved for glioblastoma multiforme, has been used with modest success in some patients [3, 5–11].

We report the fourteen-year course of a man with a large silent corticotroph adenoma who developed clinical and biochemical evidence of hypercortisolism seven years after presentation. Progressive tumor enlargement mandated multiple pituitary tumor debulking procedures and radiotherapy. Tumor enlargement occurred following the use of a glucocorticoid receptor antagonist, mifepristone. Despite courses of TMZ and carbergoline, he had further tumor progression associated with a very high Ki-67 proliferation index and developed a biopsy proven hepatic metastasis. Combination therapy with cisplatin and etoposide resulted in a substantial reduction in tumor size, near-complete regression of his liver metastasis, and dramatic decrease in ACTH secretion. This unexpectedly resulted in symptomatic secondary adrenal insufficiency.

## 2. Case Report

A 40-year old male was diagnosed with a silent ACTH-secreting pituitary adenoma in 1997 when an MRI of his sinuses demonstrated a 3 cm enhancing area of soft tissue within the inferior aspect of the sella consistent with a pituitary adenoma with no evidence of expansion. MR spectroscopy showed metabolically inert tissue. Since he was asymptomatic, he underwent active surveillance with annual MRI scans. His ACTH levels ranged between 100 and 120 pg/mL, but there was no clinical evidence of Cushing's syndrome and urine free cortisol was normal. Lab values were normal for prolactin, testosterone, LH, FSH, free T4, TSH, and alpha subunit. There was no family history of pituitary adenomas or other endocrine disorders.

**Figure 1:**
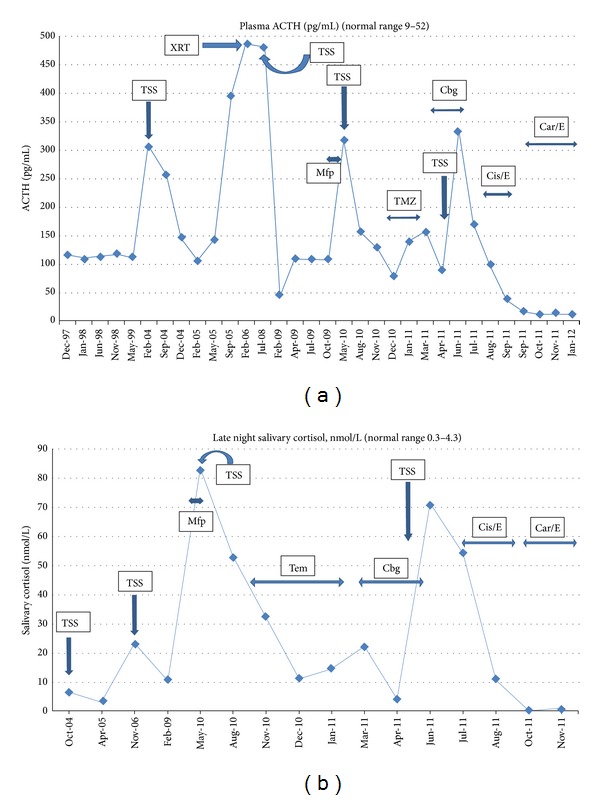
(a) Plasma ACTH levels during the clinical course. (b) Late night salivary cortisol levels during the clinical course. TSS: transsphenoidal surgery; XRT: radiation therapy; Mfp: mifepristone; TMZ: temozolomide; Cgb: carbergoline; Cis/E: cisplatin/etoposide; Car/E: carboplatin/etoposide.

**Figure 2:**
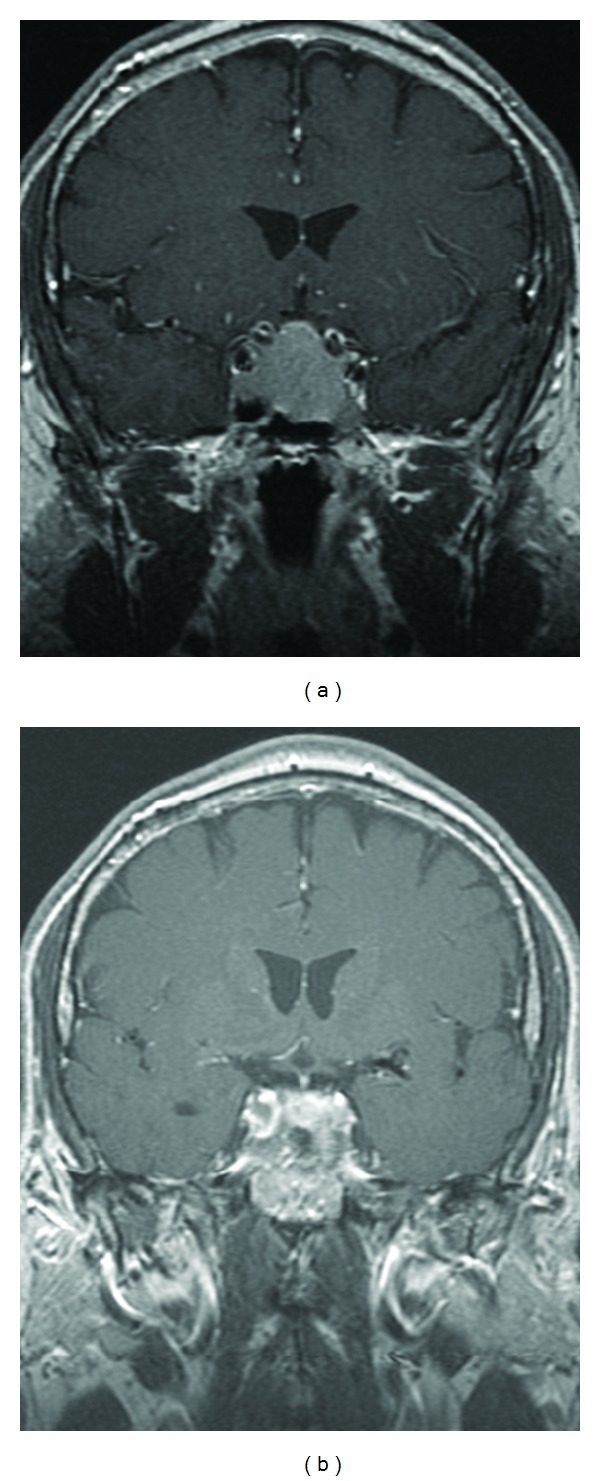
Pituitary MRI 2004. (a) Preoperative and (b) postoperative T1-weighted postgadolinium coronal images showing sellar tumor with suprasellar extension, mild mass effect on the optic chiasm, and extension into the sphenoid sinus. Postoperative image shows significant tumor debulking.

In 2004, seven years after initial discovery, his cortisol levels started to increase and he developed clinical features consistent with Cushing's syndrome including weight gain, facial rounding, hypertension, and edema. His ACTH levels increased to >300 pg/mL, which was accompanied by an increase in his late night salivary cortisol (Figure 1). An MRI showed a 2.5 × 3.3 cm enhancing mass arising in the pituitary fossa. The mass displaced the optic chiasm, invaded the sphenoid sinus, and exerted mass effect on the cavernous sinus bilaterally (Figure 2). He underwent transsphenoidal surgery (TSS) debulking of this invasive tumor. Pathology showed pituitary adenoma of corticotroph-type staining positive for ACTH and negative for TSH, prolactin, LH, GH, and FSH. Postoperative MRI showed substantial debulking with a near-complete tumor removal (Figure 2). This initially resulted in reduction of plasma ACTH to 105 pg/mL and improvement in symptoms. However, over the next year his cortisol levels continued to increase and symptoms worsened. He received adjuvant radiation therapy using a 5-field noncoplanar arrangement. A total dose of 50.4 Gy was delivered in 28 fractions over 38 days. Despite radiation therapy, his cortisol levels remained elevated. He developed radiation-induced optic neuritis manifesting with blurry vision and decreased visual acuity of the left visual field. He was treated with prednisone, pentoxifylline, and hyperbaric oxygen with improvement in his symptoms. Despite the elevated cortisol levels, his MRI over the next 2.5 years demonstrated stable disease and he developed no new symptoms.

**Figure 3:**
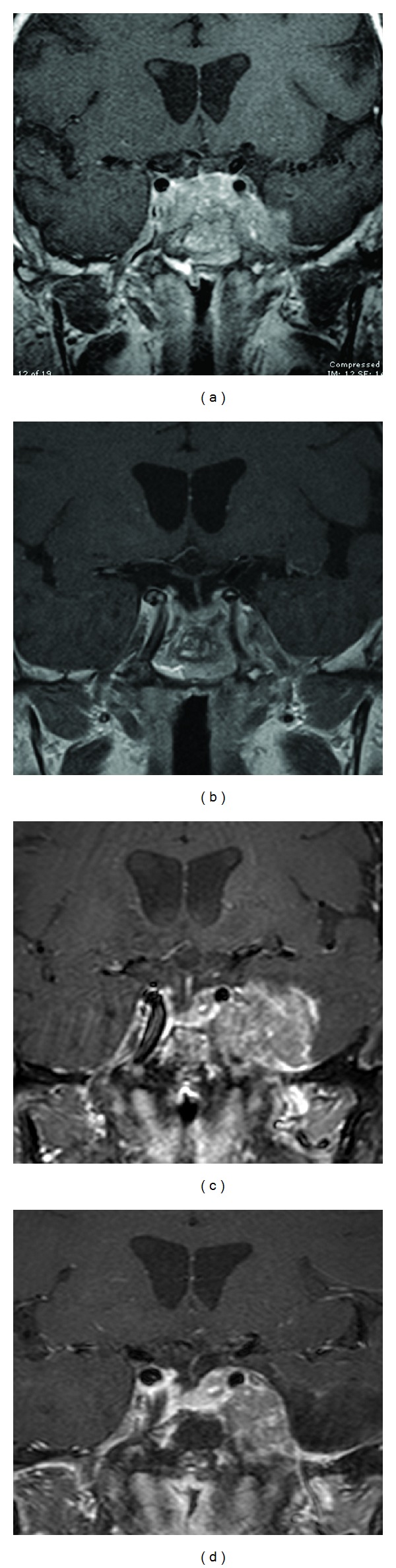
Pituitary T1-weighted postgadolinium coronal MRIs before (a) and after (b) endonasal endoscopic tumor debulking in July 2010; in (b) note decreased sellar, cavernous sinus, and Meckel's cave tumor with better visualized infundibulum and optic chiasm. Pituitary T1-weighted postgadolinium coronal MRIs before (c) and after (d) two-stage transcranial and endonasal debulking in May 2011; in (d) note decreased mass effect on left temporal lobe.

**Figure 4:**
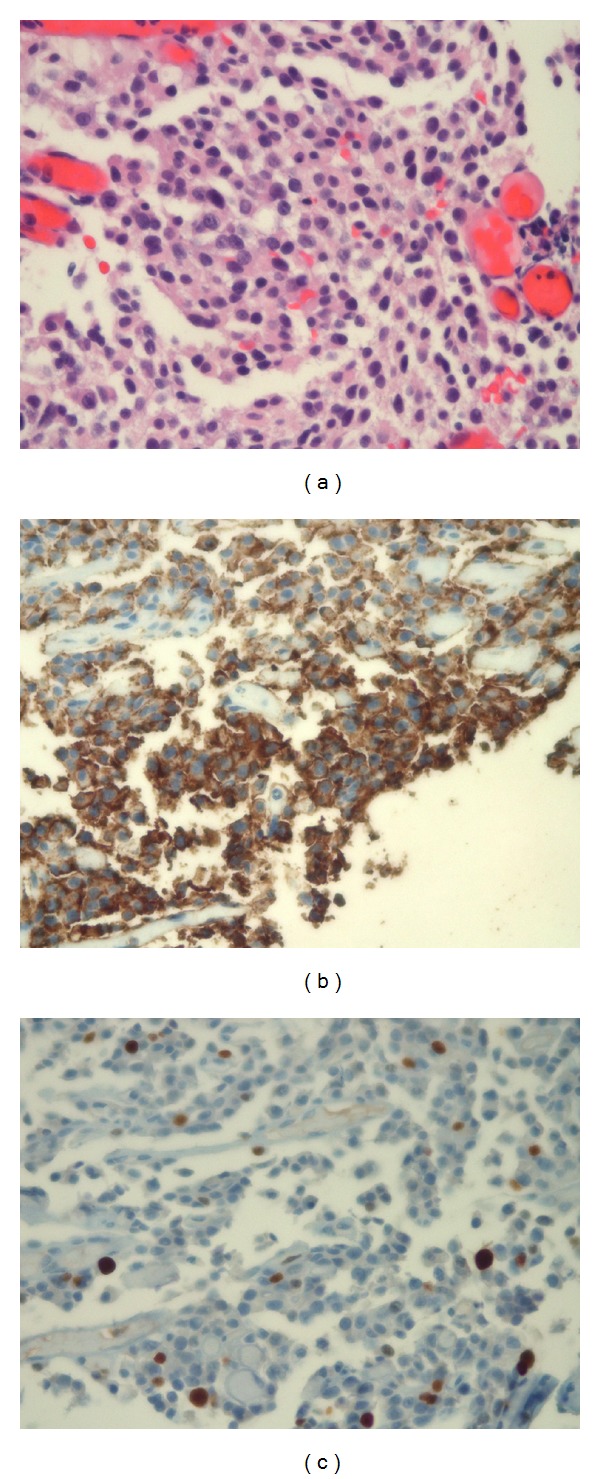
(a) H and E. The diffuse pattern of basophilic cells seen in the 2010 lesion is characteristic of corticotroph adenoma. There is mild nuclear pleomorphism. (b) ACTH stain shows positive cytoplasmic staining, confirming the diagnosis of corticotroph adenoma. (c) Ki-67, a proliferation marker, is increased to 5–7%. Values over 3% are noted as a potential indicator of more aggressive behavior but alone do not allow diagnosis as an atypical adenoma by World Health Organization criteria.

In late 2008, an MRI showed significant enlargement in mass size and he underwent another TSS tumor debulking in January 2009. Residual tumor remained predominantly in the left cavernous sinus and lateral sphenoid area. Postoperatively, late night salivary cortisol remained elevated and the patient had continued progressive signs and symptoms of hypercortisolism. He was enrolled into a clinical study with mifepristone in February 2010 (SEISMIC study). However, he experienced no clinical improvement with mifepristone which was discontinued ten weeks after initiation due to tumor enlargement. An MRI in July 2010 showed predominately left-sided extensive tumor regrowth in the sella, sphenoid sinus, and bilateral cavernous sinuses. Consequently he underwent an endonasal endoscopic tumor resection in July 2010 with substantial debulking, but significant residual tumor remained in the cavernous sinuses (Figures 3(a) and 3(b)). Pathology confirmed an ACTH-staining adenoma with a Ki-67 of 5–7% and P53 negative (Figure 4). Postoperative late night salivary cortisol and urine cortisol remained elevated. In October 2010 he was started on TMZ 200 mg/M^2^ administered orally on the first 5 days of a 28-day cycle with initial improvement in cortisol levels (Figure 1).

**Figure 5:**
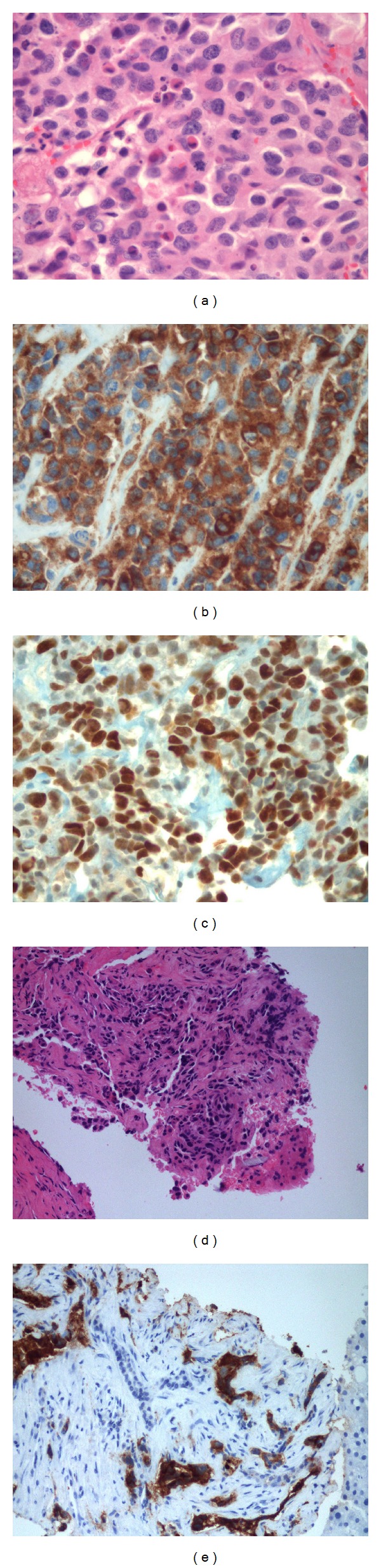
(a) H and E. The tumor in 2011 now has atypical morphologic features including increased nuclear to cytoplasmic ratios and increased nuclear pleomorphism with visible nucleoli. Numerous pyknotic cells and mitotic figures are evident indicating increased cell turnover rate. (b) ACTH stain positivity confirms this tumor as a recurrence of the corticotroph adenoma (c) Ki-67 staining is markedly increased to 80–90%. This high rate alone is not diagnostic of carcinoma but is a worrisome feature. (d) Core biopsy of liver reveals a metastatic malignancy. The morphologic appearance is not specific but is similar to the pituitary lesion. (e) ACTH positivity in the tumor cells confirms the diagnosis of metastatic pituitary carcinoma.

He was monitored and in January 2011, his ACTH levels began to increase again. Repeat MRI showed dramatic progression with tumor regrowth in the sellar and sphenoid sinus region as well as marked tumor growth in the left cavernous sinus and left middle fossa causing mass effect and edema on the left temporal lobe. TMZ was stopped and he was started on carbergoline. There was no reduction in ACTH with this treatment and it was subsequently stopped. Clinically, he had significant visual impairment and headache. Additional radiation therapy was not an option due to prior radiation exposure. In May 2011 he consequently underwent two-staged tumor debulking including a left temporal craniotomy followed 3 days later by redoendonasal endoscopic tumor debulking (Figures 3(c) and 3(d)). Pathology revealed marked tumor progression with nuclear anaplasia, numerous mitoses and dramatic increase in Ki-67 ranging from 80 to 90%; P53 remained negative; ACTH immunocytochemistry was positive (Figures 5(a)–5(c)). His postoperative brain and sellar MRI confirmed significant tumor debulking, but a restaging chest and abdominal CT showed a 16.3 mm solitary metastasis in segment five of the right hepatic lobe. This was biopsied and pathology confirmed poorly differentiated ACTH-pituitary carcinoma identical to the primary site tumor (Figures 5(d) and 5(e)).

**Figure 6:**
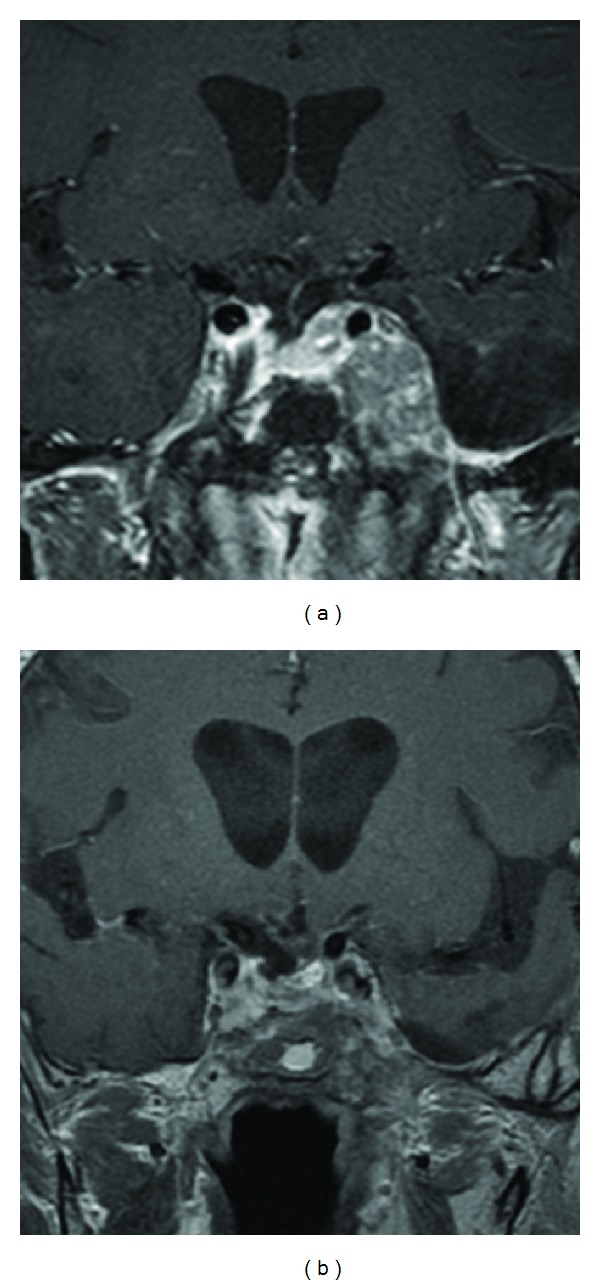
Pituitary T1-weighted postgadolinium coronal MRIs before (a) and after (b) 3 cycles of cisplatin and etoposide demonstrating significant tumor shrinkage of residual left cavernous sinus and Meckel's cave carcinoma.

Given the high proliferation index and the presence of metastatic disease, he was started on cisplatin 80 mg/M^2^ and etoposide 100 mg/M^2^ administered every 3 weeks in July 2011. Pituitary MRI after 1 cycle demonstrated interval decrease in tumor extension along the planum sphenoidale, left orbital roof, and left cranial fossa. He displayed a dramatic clinical improvement in right eye vision and headache resolution. After 3 cycles, pituitary MRI continued to show significant parasellar tumor shrinkage (Figure 6). An abdominal CT demonstrated decreased size of the liver lesion to 6 mm.

In early September 2011, he developed abrupt onset of relative hypotension, marked fatigue, and weakness. These features were consistent with acute adrenal insufficiency. Laboratory studies showed a plasma ACTH of 14 pg/mL. His basal cortisol was 0.5 ug/dL and increased to 2.6 ug/dL after the administration of cosyntropin. He had immediate clinical improvement with glucocorticoid administration. Transient adrenal insufficiency did not previously occur with any of the transsphenoidal surgeries.

After 3 cycles, the chemotherapy regimen was changed to carboplatin AUC 5 and etoposide 100 mg/M^2^ due to renal insufficiency. A total of 6 cycles was administered every 3 weeks. He had stable disease based on serial pituitary MRIs. In January 2012, he was admitted with seizures. MRI spine revealed leptomeningeal enhancement and CSF cytology was positive for malignant cells. Chemotherapy was stopped and the patient died soon thereafter. A post mortem examination was not performed.

## 3. Discussion

This report describes a man with a large silent corticotroph adenoma progressing to endogenous hypercortisolism and metastatic ACTH-pituitary carcinoma over a period of 14 years. During his treatment, multimodal therapy had variable impact on his tumor and Cushing's disease. However, he had a unique and dramatic response to cytotoxic chemotherapy with cisplatin and etoposide followed by remarkable tumor shrinkage and decrease in ACTH hypersecretion with the development of severe secondary adrenal insufficiency requiring glucocorticoid support.

The development of Cushing's disease from silent corticotroph adenomas is well appreciated. Silent corticotroph adenomas secrete precursors of ACTH that are biologically inert but are often detected in ACTH immunoassays. Changes in posttranslational processing of the ACTH prohormone POMC may result in secretion of biologically active ACTH and development of endogenous hypercortisolism. More commonly recognized now, silent corticotroph adenomas often behave aggressively. Our patient was closely observed and despite consistently elevated plasma ACTH levels, there was no evidence of hypercortisolism for seven years. It is unknown whether early intervention with surgical or radiotherapy would change the natural history of this neoplasm.

ACTH-secreting adenomas are the most common secretory pituitary tumors that undergo malignant transformation [3, 4]. The prognosis of pituitary carcinoma is generally poor, certainly once distant metastases are identified. Accordingly, clinicians have tried to identify parameters that may predict the biological behavior of pituitary tumors. Unfortunately, conventional histological evaluation is inadequate to distinguish benign from malignant tumors. Although the degree of nuclear atypia, cellular pleomorphism, and mitotic indices tend to be higher in aggressive or metastatic lesions, there is considerable overlap. Estimation of the cell cycle specific antigen Ki-67 by using the MIB-1 antibody has been shown to correlate with the invasiveness and prognosis of pituitary neoplasms [2, 19]. The MIB-1 epitope is highly repetitive and stable even after tissue fixation in formalin. Thereby, estimation of Ki-67 activity can be obtained in formalin fixed and paraffin-embedded tissue. A Ki-67 index of >3% tends to be more predictive of pituitary tumor aggressiveness and possibly malignant transformation. Nonetheless, there is considerable case-to-case variability with a wide range of Ki-67 indices in patients with pituitary carcinomas. Regardless, a Ki-67 index higher than 10% should raise suspicion for the malignant potential of a pituitary tumor [20]. Our patient had a Ki-67 index of 80–90% at the time of his final surgical debulking procedure. This very high proliferative index may have predicted the dramatic response to cytotoxic chemotherapy.

Genetic alterations involved in the development of pituitary carcinomas have also been evaluated. The quantitative immunohistologic chemical estimation of P53 expression has been thought to have some prognostic significance. Most studies have demonstrated either the appearance or increment in subsequent P53 immunostaining during the progression of a pituitary adenoma to pituitary carcinoma; however, P53 nuclear staining was absent in our patient [20, 21].

The treatment of Cushing's disease in patients with aggressive ACTH-secreting pituitary carcinomas is challenging. Pituitary-directed hormonal therapies are quite limited. Since some corticotroph tumors have dopamine 2 (D2) receptors, dopamine agonist therapy with cabergoline may demonstrate some efficacy. However, significant benefit would not be expected in high-grade poorly differentiated tumors and our patient had no effect from a course of cabergoline therapy.

ACTH-secreting carcinomas have been described in patients who have undergone bilateral adrenalectomy. Mifepristone, a glucocorticoid receptor antagonist, has been shown to produce clinical and metabolic benefits in patients with Cushing's syndrome [22]. Our patient had no clinical improvement with the use of mifepristone and after ten weeks, a slight increase in pituitary tumor size was noted and treatment was discontinued. However, any therapy that may attenuate glucocorticoid negative feedback, either by decreasing adrenal steroidogenesis or by blocking cortisol action, may potentially stimulate the pituitary corticotrophs to undergo rapid proliferation and augment neoplastic transformation.

Temozolomide, an alkylating agent FDA approved for glioblastoma, has recently been utilized for pituitary tumors refractory to standard interventions. This oral chemotherapy has 100% bioavailability with excellent CNS penetration. It is thought to have antitumor effect on malignant cells harboring low levels of DNA-repair enzyme called O6-methylguanine DNA methyltransferase (MGMT). There have been a few case reports demonstrating at least short-term success of TMZ administered with or without capecitabine as salvage chemotherapy for patients with aggressive pituitary tumors refractory to first line treatment [11, 18, 23]. After a six-month course of temozolomide, our patient had a massive progression of his pituitary tumor into the temporal lobe with subsequent development of a hepatic metastasis.

Summaries of the potential benefit of TMZ as part of management of ACTH-secreting pituitary carcinomas have been recently published [3, 18].  Table 1 summarizes the case reports in which other chemotherapy agents were administered with subsequent outcomes. In total, 9 prior detailed cases have been reported with patient ages ranging 17–59 with 3 females and 6 males.

In two cases small molecule targeted agents were administered. Everolimus, an inhibitor of the mammalian target of rapamycin (mTOR), did not induce a response and disease progression ensued [14]. In contrast to this, the monoclonal antibody bevacizumab which targets vascular endothelial growth factor (VEGF) induced a durable remission of at least 16 months [16]. In both cases, the Ki-67 index was low.

In two cases, platinum based chemotherapy was administered as combination therapy [4, 12, 18]. In both cases, this regimen type was not tolerated and patient death occurred shortly thereafter. A positive response occurred in one patient after two cycles of cisplatin and etoposide resulting in a 40% decrease in tumor volume on MRI of the brain. In this case, the Ki-67 index was high at 31% [18]. This patient also responded to a combination of capecitabine and TMZ.

Conventional chemotherapy also yielded favorable responses in two other cases [13, 15]. Gaffey et al. reported that eight cycles of cyclophosphamide, vincristine, and dacarbazine decreased ACTH levels from 3000 to 450 pg/mL. In the other case, 4 cycles of adriamycin, 5-FU, and cyclophosphamide lowered the 1800 h IR-ACTH level from 175,000 to 75,000 pg/mL. Both patients were alive at time of publication.

In the remaining cases, disease progression occurred despite the use of chemotherapy. In one case the use of TMZ monotherapy for 8 cycles followed by combination therapy with carmustine and TMZ resulted in no response. In this case, the Ki-67 index was 20% [7]. In another case, use of mitotane and carmofur resulted in progressive disease [17]. Finally, use of doxorubicin, cyclophosphamide, and etoposide was not tolerated and patient death occurred [4].

In summary, TZM as well as other chemotherapy agents can induce a response in some cases. While platinum based regimens have higher treatment related toxicities, this regimen type should be considered in select cases. The patient in this report had a Ki-67 index of 80–90% and use of platinum based chemotherapy induced a sustained remission for 6 months prior to the development of leptomeningeal disease. As with the case above, the use of cisplatin and etoposide in the setting of a Ki-67 index of 31% also decreased tumor burden [18]. Therefore, patients with good functional status and aggressive tumors, evidenced by high Ki-67, should be considered for conventional cytotoxic platinum based chemotherapy in the appropriate clinical setting.

Pasireotide is a new somatostatin analog with a unique, broad somatostatin-receptor-binding profile. It has high affinity for the somatostatin-receptor subtype 5 that predominates in ACTH-secreting tumors. Pasireotide has been shown to decrease cortisol levels and reduce tumor size in patients with Cushing's disease. Although pasireotide is FDA-approved for the treatment of hypercortisolism due to corticotroph adenomas, it was not available at the time when this patient was treated and there are no reports of its efficacy in patients with ACTH-secreting pituitary carcinomas [24].

It is well appreciated that poorly differentiated, high-grade anaplastic neuroendocrine tumors exhibiting high proliferative indices, such as small cell lung cancer, may response well to cisplatin and etoposide. The response to these agents in our patient was gratifying and dramatic. There was a significant reduction of tumor burden shortly after introduction of cisplatin and etoposide with prompt improvement in visual impairment as well as significant regression of the hepatic metastasis.

Three months after introduction of chemotherapy, the patient had a rapid reduction in ACTH and cortisol levels. He then presented with relatively acute onset overwhelming fatigue, weakness, and relative hypotension. Biochemical studies confirmed the presence of hypocortisolism and a low plasma ACTH. Glucocorticoid support with prednisone was provided with a rapid clinical improvement. To our knowledge, this is the first report of chemotherapy-induced regression of an ACTH-secreting pituitary carcinoma accompanied by secondary adrenal insufficiency.

Six months after initiation of chemotherapy he was diagnosed with leptomeningeal disease from his malignancy. Unlike TZM, cisplatin and etoposide have variable penetration into the CNS based on the patency of the blood brain barrier. The development of leptomeningeal disease may have been secondary to inadequate chemotherapy penetration to all parts of the CNS. Alternatively, it is possible that progression was secondary to chemotherapy resistance. Ultimately, the patient declined any further chemotherapy.

In summary, silent corticotroph adenomas may become clinically active and associated with hypercortisolemia and malignant transformation. Since ACTH-secreting pituitary carcinomas have many similarities to other neuroendocrine tumors, chemotherapy with cisplatin and etoposide may provide significant clinical benefit, particularly in those tumors with high proliferative indices. Given the potential dramatic responses from such chemotherapy agents, the development of secondary adrenal insufficiency may occur and patients should be monitored accordingly. As a means to better personalize therapy, further inquiry into the molecular characterization of pituitary carcinomas is warranted. In addition, this case is a summary of other cases in which chemotherapy agents other than TZM have been utilized.

## Figures and Tables

**Table 1:** Chemotherapy in metastatic pituitary corticotroph carcinomas.

Author, year of publication	Age	Gender	Metastatic sites	Chemotherapy	Chemotherapy cycles/duration, doses, and response	Treatments prior to chemotherapy	Ki-67	Patient outcome
Farrell et al., 2003 [12]	34	F	Pelvic, vertebrae, and ribs	VCR, carboplatin, and etoposide	Not tolerated	TSS, XRT, metyrapone, ketoconazole, SR, TMZ, bADX, and OCT	3%	Death from progression
Gaffey et al., 2002 [13]	59	F	Liver	CTX, VCR, and dacarbazine	8 cycles lowered ACTH from 3000 pg/mL to 450 pg/mL	TSS, bADX, XRT, SR, and OCT	NR	Alive at publication
Jouanneau et al., 2012 [14]	45	M	Craniocervical	Everolimus	3 months, progressive disease	TMZ, pituitary surgery, SR, XRT, and bADX	Low	Death from progression
Kaiser et al., 1983 [15]	17	F	Liver, lung, pelvis, vertebrae, and mediastinum	Adriamycin, 5-FU, and CTX	4 Cycles lowered 1800 h IR-ACTH from 175,000 pg/mL to 75,000 pg/mL	Pituitary surgery, XRT, and bADX	NR	Alive at publication
Moshkin et al., 2011 [16]	38	M	Vertebrae	Bevacizumab	16 months, stable disease	TSS, XRT, and TMZ	1–5%	Alive at publication
Nawata et al., 1990 [17]	53	M	Liver, lung, and brain	Mitotane and HCFU	Progressive disease	Pituitary surgery, XRT		Death from progression
Raverot et al., 2010 [7]	31	M	NR	BCNU and TMZ	6 cycles of combination therapy, progressive disease	TSS, SR, TMZ, and bADX	20%	NR
Thearle et al., 2011 [18]	50	M	Bone, vertebrae	Capecitabine and TMZ Cisplatin and etoposide	4 cycles lowered ACTH from 1874 pg/mL to 85 pg/mL 2 cycles, 40% decrease in tumor volume on MRI brain	TSS, gamma knife, ketoconazole, metyrapone, OCT, bADX, cabergoline, and rosiglitazone	31%	Enrolled in hospice after 2 cycles of cisplatin and etoposide
Van der Klaauw et al., 2009 [4]	23	M	Bone, liver	Doxorubicin, CTX, and etoposide	2 cycles, not tolerated	XRT, ketoconazole, TSS, brachytherapy, bADX, and OCT	NR	Death from progression

bADX: bilateral adrenalectomy; BCNU: carmustine; CTX: cyclophosphamide; DA: dopamine agonists; 5-FU: fluorouracil; HCFU (carmofur): 1-hexylcarbamoyl-5-fluorouracil; OCT: octreotide; SA: somatostatin analogs; SR: stereotactic radiosurgery; TMZ: temozolomide; TSS: transsphenoidal surgery; VCR: vincristine; XRT: external beam radiation therapy; NR: not reported; IR-ACTH: immunoreactive ACTH (normal range 5–45 pg/mL at 1800 h).
